# Influence of Zinc Doping on the Morphological, Structural, and Optical Characteristics of Copper Oxide Thin Films Prepared Through Ultrasound Spray Pyrolysis

**DOI:** 10.3390/ma19081596

**Published:** 2026-04-15

**Authors:** Isis Chetzyl Ballardo Rodríguez, Brahim El Filali, Aarón Israel Díaz Cano, Rebeca Jiménez Rodríguez, Juan Antonio Jaramillo Gómez

**Affiliations:** 1 Instituto Politécnico Nacional, UPIITA, Mexico City 07340, Mexico; iballardor@ipn.mx (I.C.B.R.); jjaramillo@ipn.mx (J.A.J.G.); 2 Instituto Politécnico Nacional, SEPI-ESIME-Zac, Mexico City 07320, Mexico; 3 Instituto Politécnico Nacional, SEPI-UPIITA, Mexico City 07340, Mexico; aidiaz@ipn.mx; 4 Instituto Politécnico Nacional, ESIME-Zac, Mexico City 07320, Mexico; rjimenezr1001@alumno.ipn.mx

**Keywords:** Zn_x_Cu_1−x_O alloy, Zn doping, band gap XRD, SEM

## Abstract

The study of wide-bandgap nanomaterials has gained considerable attention in recent years, especially in the case of semiconductor oxides that exhibit full or partial optical transparency in fundamental research and technological applications. These include optoelectronic devices, gas sensors and photovoltaic cells, among others. The activation or adjustment of optical and structural properties, especially the bandgap and the parameters of unit cell lattice, can be achieved by varying the dopant concentration during the synthesis of semiconductor thin films in these applications. In this context, copper oxide has emerged as a valuable material, owing to its thoroughly analyzed structural behavior and its broad potential across multiple technological fields. The present work focuses on the synthesis of zinc-doped copper oxide (Zn_x_Cu_1−x_O) thin films on silicon and quartz substrates through ultrasonic spray pyrolysis. The effects of varying the zinc doping concentration (0.0, 5.0, 10.0 and 20.0 at. %) on the morphological, structural, and optical characteristics of the Zn_x_Cu_1−x_O films were analyzed. Scanning electron microscopy (SEM) analysis indicated a gradual increase in nanoparticle size, rising from 221 nm for CuO to approximately 322 nm for the Zn_0.2_Cu_0.8_O samples as the zinc content increased. Structural characterization via X-ray diffraction (XRD) confirmed a monoclinic crystal arrangement belonging to the $C_{2h}^{6}$ (c2/c) space group. As the percentage of zinc increased, the XRD peaks shifted to lower angles, consequently increasing the volume and crystal lattice parameters of the Zn_x_Cu_1−x_O structure; this finding was additionally supported by a redshift observed in the Raman analysis. The transmittance spectra of the films showed low transmittance between 40 and 44%. The optical bandgap of the Zn_x_Cu_1−x_O thin films was estimated from the transmittance data by applying the Tauc plot method. A decrease in the band gap was observed at higher doping concentrations. It can be confirmed that no secondary phases are observed at a doping level of 20.0 at. % of zinc, indicating good solubility of zinc in CuO. The analysis and discussion of these findings are included throughout this work to elucidate the controversies noted in the literature.

## 1. Introduction

Global warming and its various known negative effects have generated significant attention within the international scientific community toward renewable energy sources, including biofuels, hydroelectric, geothermal, and wind energy, as well as solar energy [1]. Solar energy is the most economical, reliable, and relatively simple to generate. One of the most important components in transparent devices such as solar cells, batteries [2], and optoelectronic devices [3], among others, are the transparent semiconductor oxides used to form front- and back-side contact electrodes [4].

Copper oxide (CuO) has established itself as one of the attractive p-type semiconductors, characterized by its moderate band gap (1.2–1.9 eV) [5], greater physicochemical stability, as well as photovoltaic, electrochemical, catalytic properties and high visible light absorption. Unlike many oxide-based semiconductors, copper oxide is characterized by its low synthesis cost and robust environmental stability, making it an attractive candidate for sustainable technologies such as photovoltaic panels, photocatalysis, and especially transparent electronics applications [6]. Because copper oxide is a non-toxic material, it has generated increasing demand for environmentally friendly functional materials, particularly in consumer electronics, medical applications [7,8] and for removing toxic pollutants [9,10].

The use of copper oxide in these applications depends primarily on its microstructure, which is closely related to the synthesis techniques. The quality of the crystalline, grain orientation and morphology, and surface area of the semiconductor thin films are means of controlling the properties, primarily the optoelectronic properties, of the resulting devices. Various CuO thin film synthesis techniques have been reported in the literature. These fabrication methods have advantages and limitations. For instance, vacuum-based deposition techniques like thermal evaporation [11] and magnetron sputtering [12] produce high-quality and high-purity films, but are also expensive and have limited scalability. In contrast, spin coating produces uniform films [13], and dip coating allows for controlled film thickness through iterative cycles. Spray pyrolysis allows the control of the film thickness [14,15], the ability to cover large areas, and reduced costs due to its simple setup and minimal material consumption [16,17].

Controversy exists concerning the impact of Zn metal ion doping on the bandgap and nanocrystalline size. For instance, in articles [1,18], the authors indicate that the energy gap rises from 2.8 to 2.94 eV as the percentage of zinc ion doping increases. Conversely, in article [19], it is reported that the energy gap and particle size decrease within the zinc doping range of 0–3%, followed by an increase in the 3–5% doping range. However, the articles discussed do not provide a satisfactory explanation for this observation.

This investigation addresses the optical and structural analysis and study of Zn_x_Cu_1−x_O thin films obtained by spray pyrolysis. The analysis reveals how the percentage of Zn refines the grain size and influences the band gap. This work not only delves into the fundamental understanding of the Zn_x_Cu_1−x_O alloy but also provides practical insight for obtaining this type of thin film.

## 2. Materials and Methods

The copper (II) nitrate hydrate (Cu(NO_3_)_2_ 3H_2_O) served as the copper source for preparing the primary solution, while the zinc acetate dihydrate (Zn(CH_3_COO)_2_ 2H_2_O) functioned as the zinc dopant source. All used chemicals are high-purity (≥99%) products from the Sigma Aldrich brand (St. Louis, MO, USA). Both solutions were formulated at a molar concentration of 0.12 M, utilizing an 8:1:1 ratio of methanol, distilled water, and acetic acid. Continuous stirring was employed to ensure thorough mixing. The addition of acetic acid contributed to the solutions’ stability, yielding clear and homogeneous mixtures with a pH of 6. Following this, the two solutions were combined in predetermined ratios to achieve zinc concentrations of 0.0, 5.0, 10.0, and 20.0 at.%. Prior to the synthesis process, the silicon substrates underwent treatment with 30% nitric acid to eliminate oxides. Following this, both the silicon and quartz substrates were subjected to a 30 min cleaning in an ultrasonic bath filled with distilled water. They were then cleaned in succession with acetone, distilled water, and methanol, and ultimately dried using a nitrogen flow. The precursor solution was then deposited onto the substrates via ultrasonic spray pyrolysis at 350 °C over a period of 70 min, maintaining a nitrogen carrier flow of 4 L/min. To enhance their crystallinity, the deposited films underwent heat treatment in an uncontrolled oxygen-rich environment at a temperature of 350 °C for two hours.

Superficial morphology of the Zn_x_Cu_1−x_O thin films was examined by scanning electron microscopy (SEM) using the JEOL JSM-7800F equipment (JEOL, Tokyo, Japan). X-ray diffraction (XRD) patterns were recorded with an X’ Pert MRD diffractometer (Malvern Panalytical, Worcestershire, UK) featuring a copper X-ray source (Kα_1_ = 1.5406Å). Transmittance measurements were performed using a USB2000 UV-VIS spectrometer from Ocean Optics (Orlando, FL, USA), which was equipped with a DT 1000 CE US.VIS lamp. The Job-Yvon Lab RAM HR 800UV micro-Raman (Horiba, Kyoto, Japan) system was utilized to observe Raman scattering spectra excited by a wavelength of 532 nm.

## 3. Results and Discussion

### 3.1. X-Ray Diffraction Analysis

**Figure 1:**
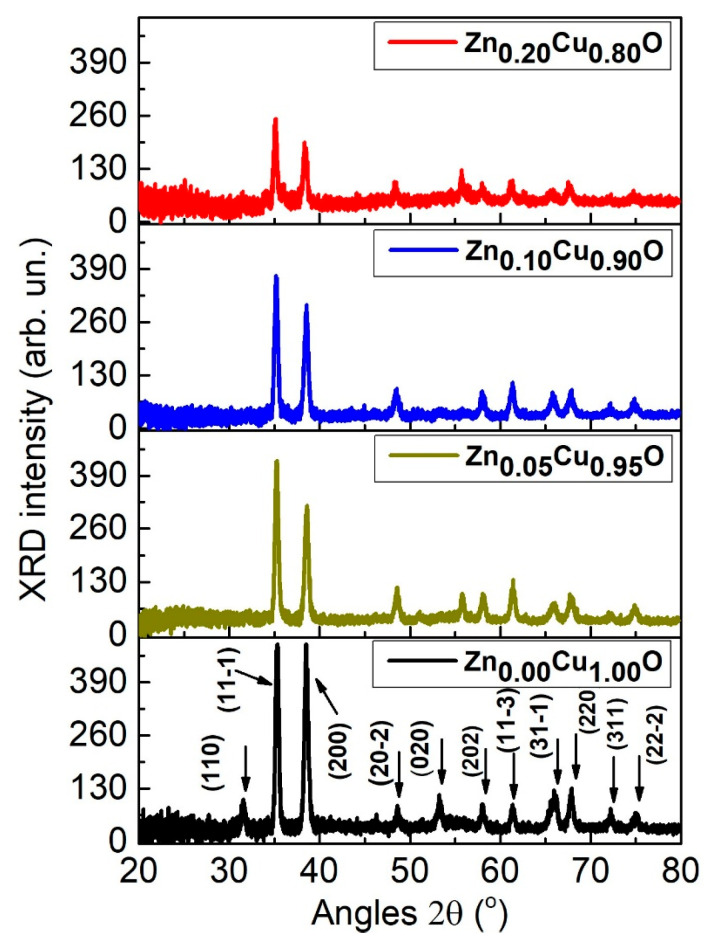
XRD spectra of Zn_x_Cu_1−x_O thin films.

The X-ray diffraction (XRD) spectra shown in Figure 1 of the Zn_x_Cu_1−x_O samples clearly demonstrates the polycrystalline nature of our films synthesized using the ultrasonic spray pyrolysis technique. The X-ray diffraction peaks detected for the Zn_x_Cu_1−x_O samples are: (11-1), (200), (20-2), (021), (202), (11-3), (31-1), (220), (311) and (22-2) which correspond to the monoclinic crystal system of CuO (JCPDS-062-8615), at the percentages 0.0, 5.0 and 10.0 at.% of Zn. No secondary phases or impurities are detected.

For all samples, the characteristic Cu_2_O peak at the 2θ angle, centered at approximately 36.4°, was absent. It should be noted that this peak is present in most crystallographic charts (JCPDS-00-0030898, JCPDS-00-001-1142, JCPDS-00-003-0892, JCPDS-00-003-0898). In fact, this peak has been reported in several publications on the synthesis of thin CuO films [20,21]. The presence of this Cu_2_O peak in CuO films can be attributed to several factors, including the synthesis temperature, the thermal treatment temperature, and the amount of oxygen present during the same process. In our case, the film synthesis as well as the treatment were done in an oxygen-rich environment. These conditions appear to have been ideal for obtaining thin CuO films without the presence of intermediate cuprous oxide or other secondary phases. Analyzing the spectra presented in Figure 1, it can be confirmed that these are polycrystalline thin films with a preference for the (11-1) and (200) planes. The XRD intensity decreases as x content dopant increases, with the lowest intensity occurring at x = 0.20, indicating the beginning of the unit cell destruction process. With increasing percentages of Zn in the Zn_x_Cu_1−x_O (x = 0.0, 0.05, 0.10 and 0.20) thin films, a shift towards smaller angles was observed (Table 1). This effect is due to the slight difference in ionic radio, with Zn^2+^ (0.74 Å) being larger than the host Cu^2+^ ion (0.73 Å).

In the doped Zn_x_Cu_1−x_O samples, no secondary phase is present, indicating that a homogeneous mixture was obtained where the Cu ion was replaced by the Zn ion. The larger radius of the Zn ion in comparison to the Cu ion resulted in a small displacement at low angles (Table 1). According to Bragg’s law equation $\left(n\lambda =2d_{\mathrm{hkl}}\sin\left(\theta \right)\right)$ [22], this will lead to an increase in the unit cell volume.

### 3.2. Scanning Electron Microscopy

**Figure 2:**
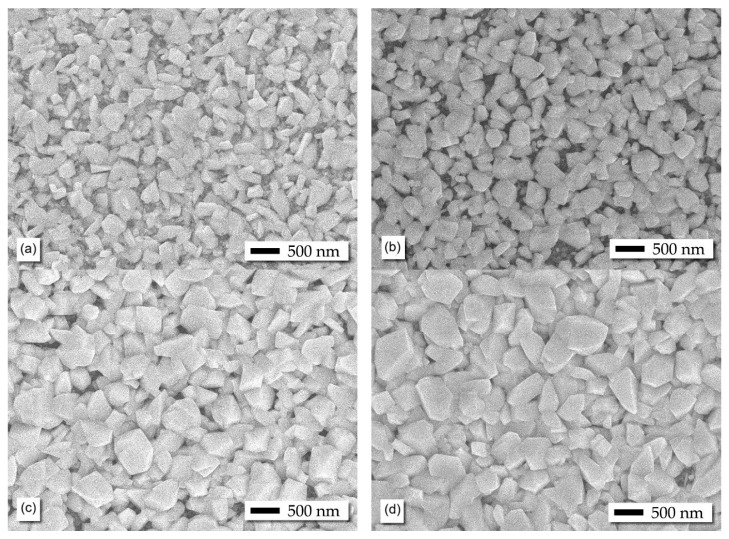
SEM images of Zn_x_Cu_1−x_O thin films with varying Zn concentrations: (**a**) 0.0 at%., (**b**) 5.0 at%., (**c**) 10.0 at%. and (**d**) 20.0 at%.

The SEM images presented in Figure 2 show how the surface morphology of the Zn_x_Cu_1−x_O thin films evolves with the percentage of Zn. For the samples with 0.0 and 5.0 at.% Zn, the surface is composed of grains distributed in a geometrically cubic pattern that is not regular in shape and size, and exhibits greater irregularity, with an average size of approximately 221 ± 30 nm for the CuO sample and 274 ± 17 nm for the Zn_0.05_Cu_0.95_O sample. Both samples have a similar surficial area, primarily due to their rough and porous appearance.

In the Zn_0.10_Cu_0.90_O and Zn_0.20_Cu_0.80_O samples, the nanocrystals increase slightly in size, with more uniform grains in both size and shape, although the grain size for the 10.0 at. % Zn is smaller compared to that of the 20.0 at. % Zn. The average crystallite size was estimated at 322 ± 18 nm for Zn_0.10_Cu_0.90_O and 340 ± 19 nm for Zn_0.20_Cu_0.80_O. It is observed that the porosity and superficial area were decreased especially for the Zn_0.20_Cu_0.80_O sample. For both samples, it was detected that the nanocrystals are densely packed, with little space between them.

Several publications in the literature [19,23] indicate that obtaining uniform thin films of Zn_x_Cu_1−x_O is not a simple task. To achieve this, in our case, a seed layer was placed on the substrate before starting the film deposition process to ensure uniformity. It was noted that in the synthesized samples, an increase in the doping percentage corresponded with a larger size of the nanocrystals. This behavior may be ascribed to the difference in ionic radii between Zn^2+^ (0.74 Å) and Cu^2+^ (0.73 Å), with the former being slightly larger. This finding was corroborated by X-ray diffraction analysis.

### 3.3. Raman Spectroscopy

**Figure 3:**
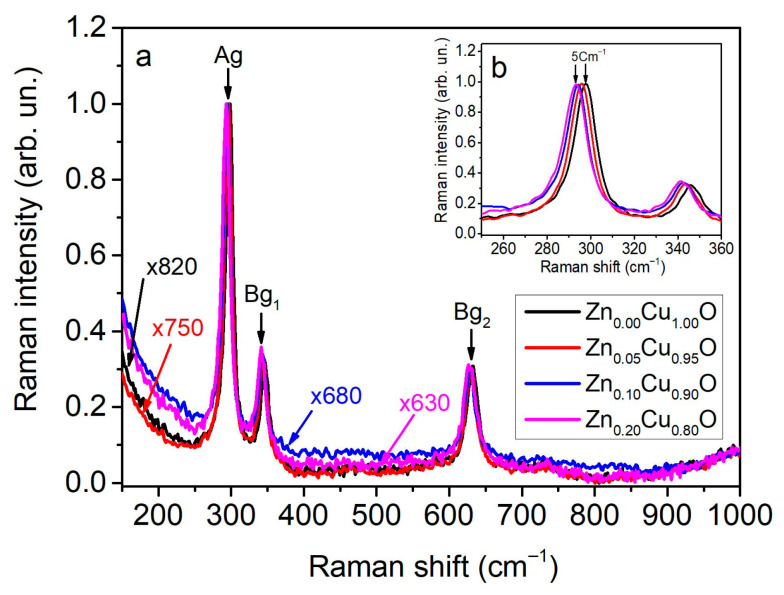
(**a**) Raman spectra of Zn_x_Cu_1−x_O thin films, (**b**) enlarged view of the region 250–360 cm^−1^.

The structural properties of nanocrystalline samples can be effectively investigated through Raman spectroscopy, which provides insight into the vibrational modes associated with the crystal structure. Raman spectra obtained at room temperature for the Zn_x_Cu_1−x_O samples are shown in Figure 3. The copper oxide semiconductor material is of the tenorite phase and belongs to the $C_{2h}^{6}$ (c2/c) space group; it also has a monoclinic crystalline phase.

Copper oxide contains four molecules within its crystallographic unit cell and two molecules in the primitive unit cell [24,25], amounting to a total of four atoms (3N). Consequently, this leads to twelve vibrational modes: three of these are Raman-active modes (Ag + 2Bg), six are IR-active modes (3Au + 3Bu), and three are acoustic modes (Au + 2Bu) [26].

The vibrations of oxygen atoms give rise to the Raman-active modes in the CuO semiconductor, while the copper atoms remain fixed in place. The Raman-Ag-active mode is linked to the out-of-phase or in-phase rotation of copper oxide, whereas the Bg_1_ mode is related to the bending of CuO, and the Bg_2_-active mode corresponds to the symmetric stretching of oxygen within the CuO unit cell [26,27].

For the undoped CuO sample, the following Raman-active modes were detected: centered at 298 cm^−1^, corresponding to Ag, which is associated with the Cu and O atoms vibrations; and the Bg-active mode with frequencies centered at 347 and 632 cm^−1^. As zinc dopant atoms were introduced into the CuO lattice, a redshift of about 5 cm^−1^ (Figure 3b) was observed in the Ag and Bg modes as the doping level increased from 0% to 20 at. %, while the intensity of the Ag and Bg_1_ peaks decreased. The redshift of the Raman-active peaks is attributed to the increased size of the Zn_x_Cu_1−x_O nanocrystals.

In the Raman spectra Figure 3, the peaks for the Zn_0.20_Cu_0.80_O sample were located at 293, 343, and 633 cm^−1^, corresponding to Ag, Bg_1_, and Bg_2_, respectively (Figure 3). This result is confirmed by SEM analysis, which revealed the presence of defects, expansion of the CuO unit cell, and a red shift of the Ag mode due to the doping level, with a Zn ion radius slightly larger than that of a Cu ion [28].

The redshift of the Bg_2_ mode is primarily due to relaxation in the unit cell because of O–O bond stretching [14]. This result agrees with the increased unit cell volume observed in XRD. No additional peaks corresponding to secondary phases were detected in the Raman spectra, confirming that all samples share the same crystal structure [25,27].

### 3.4. Transmittance Spectra and Band Gap Energy

**Figure 4:**
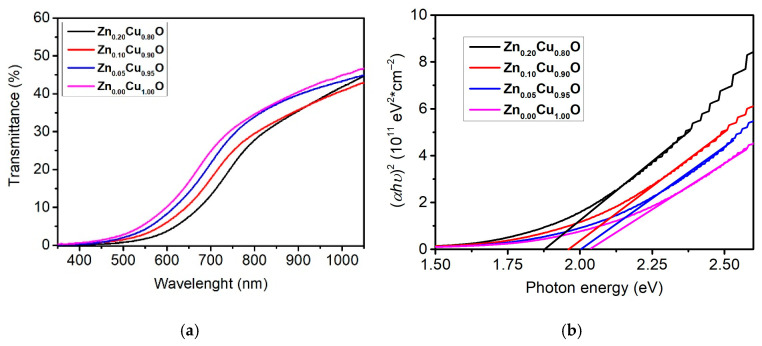
(**a**) transmittance spectra and (**b**) Tauc plot of prepared Zn_x_Cu_1−x_O thin films.

Transmittance spectra for undoped and Zn-doped CuO thin films are shown in Figure 4. Measurements were taken in the wavelength range of 350 to 1050 nm. All films exhibited low transmittance between 40 and 44% at 1050 nm. Despite increasing the doping level, the transmittance did not change significantly. This result may be related to the absence or lack of ZnO formation in our films. This finding was also confirmed by XRD analysis.

Similar results have been documented in the literature recently, although not necessarily at the same level of doping. However, the same trend in transmittance is reported for Zn_x_Cu_1−x_O [28,29]. It can be observed that the transmittance reaches very close to 0% for wavelength values between 200 and 500 nm [29,30].

For determining the optical band gap for the synthesized nanostructures, the Tau equation is given by [31]:

(1) $${\left(\alpha h\vartheta \right)}^{n}=A\left(h\vartheta -E_{g}\right),$$

where “α” is the absorption coefficient, “hϑ” is the photon energy, “A” is an energy-independent constant, and E_g_ is the optical band gap energy. The value of “n” is 2 for a direct band gap and 1/2 for an indirect band gap. The optical band gap energies were determined from plots of ${\left(\alpha h\vartheta \right)}^{2}$ versus hϑ, extending the linear portion ${\left(\alpha h\vartheta \right)}^{2}$ of the curves to the energy axis; Figure 4 illustrates the corresponding results.

By employing spectra transmittance measurements and the Tauc equation [32], the band gap values Eg were determined, as shown in Figure 4b. With a doping percentage of 0.0% at. Zn, the average band gap value was determined to be 2.05 eV. For doping percentages of 5.0, 10.0, and 20.0% at., the band gap values were 2.00 eV, 1.94 eV, and 1.88 eV, respectively.

The results reflected in the XRD analysis showed an increase in the lattice parameters of Zn_x_Cu_1−x_O. The SEM image analysis revealed that the nanoparticle size increased with increasing Zn dopant content. These results are consistent with the inverse relationship between the band gap and the doping level. All transmittance results (Eg calculations) are consistent with the SEM and XRD studies. The increase in lattice parameters in the XRD analysis is explained by the larger radius of the Zn ion compared to the Cu ion, while the decrease in the band gap can be explained by the quantum confinement equation, where the increased nanoparticle radius leads to a decrease in the band gap [22,33]. It is extremely important to mention that the results obtained from Zn_x_Cu_1−x_O (x = 0.0, 0.05, 0.10 and 0.20) semiconductor thin films grown by the spray pyrolysis technique coincide with several of the results documented in the literature.

## 4. Conclusions

Zinc-doped copper oxide thin films were produced through spray pyrolysis. The impact of varying the zinc percentage on the structural, optical, and morphological properties of the Zn_x_Cu_1−x_O thin semiconductor films was investigated. The semiconductor films exhibited a monoclinic polycrystalline structure without any defined preferential orientation. XRD spectra showed a slight shift towards smaller angles with increasing Zn doping percentage, indicating an increase in the CuO unit cell parameters. Furthermore, the intensity of the X-ray diffraction patterns decreased sharply, especially for the Zn_0.20_Cu_0.80_O sample, thus indicating the onset of unit cell destruction. No secondary phase was observed, indicating that up to a Zn percentage of 20.0 at. %, zinc remains soluble in CuO, at least under the growth conditions and heat treatment of our samples. SEM images and the corresponding nanoparticle size distribution revealed a gradual increase in the average nanocrystal size. The transmittance spectrum revealed close transparency levels, practically reaching 42% in the near-infrared spectral range for the studied films. Furthermore, with Tauc equation we detected a decrease in the band gap, ranging from 2.05 eV for Zn_0.00_Cu_1.00_O to 1.88 eV for Zn_0.20_Cu_0.80_O. Raman spectra showed a redshift in the Ag and Bg modes. These results are attributed to the increased lattice parameters, confirmed by X-ray diffraction analysis, and the increased average nanoparticle size of the Zn_x_Cu_1−x_O films, due to the increased penetration of zinc ions into the unit cell of the CuO thin films.

## Figures and Tables

**Table 1:** The variation in XRD peak positions of Zn_x_Cu_1−x_O films with Zn contents.

Sample	(110)	(11-1)	(200)	(20-2)	(020)	220
Zn_0.00_Cu_1.00_O	31.61	35.40	38.64	48.72	53.48	67.88
Zn_0.05_Cu_0.95_O	--	35.29	38.61	48.66	53.44	67.80
Zn_0.10_Cu_0.90_O	--	35.25	38.58	48.56	53.37	67.72
Zn_0.20_Cu_0.80_O	--	35.15	38.42	48.42	--	67.60

## Data Availability

The original contributions presented in this study are included in the article. Further inquiries can be directed to the corresponding author.
